# A Case of Acute Esophageal Necrosis from Unruptured Thoracic Aortic Aneurysm

**DOI:** 10.1155/2020/3575478

**Published:** 2020-05-29

**Authors:** Abhishek Polavarapu, Dhineshreddy Gurala, Bindu Mudduluru, Pretty Sara Idiculla, Jobin Philipose, Magda Daoud, Naureen Narula, Vivek Gumaste

**Affiliations:** ^1^Gastroenterology and Hepatology, Staten Island University Hospital, Northwell Health, Staten Island, New York, USA; ^2^Internal Medicine, Staten Island University Hospital, Northwell Health, Staten Island, New York, USA; ^3^Medicine, Sree Gokulam Medical College and Research Foundation, Trivandrum, India; ^4^Department of Pulmonary and Critical Care, Staten Island University Hospital, Northwell Health, Staten Island, New York, USA; ^5^Department of Gastroenterology and Hepatology, Richmond University Medical Center, Staten Island, New York, USA

## Abstract

Acute esophageal necrosis (AEN), also known as black esophagus due to its appearance on endoscopy, classically involves the distal esophagus (97% of cases). AEN affecting the midesophagus with sparing of the distal esophagus is rare and usually occurs in patients with thoracic aortic aneurysmal (TAA) rupture or aortic dissection. Herein, we report a unique case of AEN in the midesophagus in a patient with an unruptured and undissected TAA.

## 1. Introduction

AEN is also known as the “black esophagus” caused predominantly by hypoperfusion of esophagus and characterized by diffuse, circumferential, black-appearing distal esophageal mucosa that stops at the gastro-esophageal junction (GEJ) but can extend proximally to various lengths on esophago-gastro-duodenoscopy (EGD) [1, 2]. It is a rare disease, and prevalence was 0.001% to 0.2% in a retrospective endoscopy case series [3]. It is more common in older men than women with a ratio of 4 : 1 and a mean age of 68. Its prevalence is higher in people with underlying medical conditions like hypertension, diabetes mellitus, renal insufficiency, Pulmonary diseases, and cardiac diseases [2]. The esophagus is an expandable muscular organ connecting the pharynx with the stomach with a diverse blood supply. The upper esophagus derives its blood supply from descending branches of the inferior thyroid artery. Midesophagus is supplied by the bronchial and esophageal branches of the thoracic aorta. The distal esophagus is supplied by the branches of the left gastric and left inferior phrenic artery and is the least vascular compared to the proximal and middle esophagus, which makes it slightly more watershed and more susceptible to ischemic injury [2]. The etiology of AEN is multifactorial and results from a combination of tissue hypoperfusion, impaired local defense barriers, and the massive influx of gastric contents that overwhelm the esophageal mucosa [2]. Hypoperfusion can arise from a variety of causes such as acute blood loss, trauma, shock, sepsis, and congestive heart failure, which makes the distal esophagus more vulnerable to injury. Other risk factors include infections, vascular diseases, diabetes mellitus, medications, advanced malignancies, and malnutrition [2, 4–6]. Proposed pathophysiology has two hypotheses: the initial event is the low-flow vascular state and defect in mucosal system repair (as seen in malnourished and debilitated physiological states) which predisposes the esophageal mucosa to a severe topical injury. Upper gastrointestinal (GI) bleed is the most common clinical presentation of AEN in approximately 90% of patients, with or without hemodynamic compromise. Other symptoms include epigastric pain, chest pain, dysphagia, and signs of sepsis, including tachycardia and hypotension [7]. We report a case of AEN in the midesophagus in a critically ill patient with unruptured and undissected TAA.

## 2. Case Description

**Figure 1:**
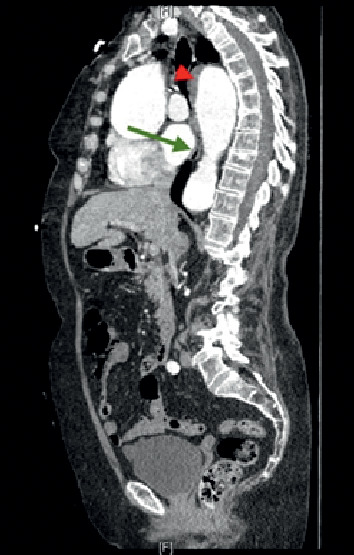
Sagittal section of CT angiogram of the chest showing thoracic aorta aneurysm of approx. 5.5 cm size causing extrinsic compression at midesophageal level (green arrow) along with intramural thrombus in the thoracic aorta (red arrowhead).

A 79-year-old female presented to the emergency department with near syncope. She denied chest pain or gastrointestinal symptoms. She had a history of hypertension, atrial fibrillation on coumadin and TAA of 5.5 cm in size, diagnosed one month prior, and scheduled for repair. Her vital signs were stable with a blood pressure of 122/62 mmHg and a heart rate of 81 beats/minute, afebrile. Her initial laboratory studies were stable except for elevated d-dimer of 2029 and subtherapeutic international normalized ratio (INR) of 1.1. Physical examination was positive for bibasilar crackles on auscultation of lung fields. Computed tomography angiogram (CTA) of the chest revealed an acute bilateral pulmonary embolism (PE) with stable TAA and mural thrombus in descending thoracic aorta (Figure 1). Echocardiogram showed evidence of right heart strain, and she was started on heparin.

**Figure 2:**
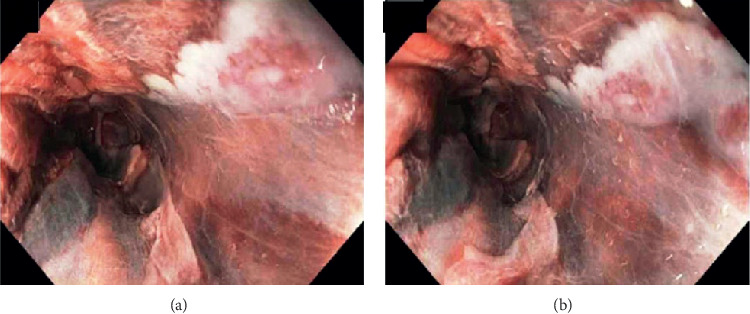
Endoscopic images showing extensive circumferential esophageal ulcerations with blackish discoloration.

**Figure 3:**
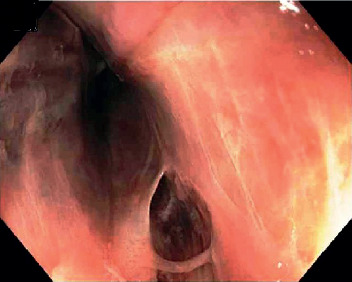
Endoscopic image showing friable esophageal mucosa, mucosal separation without active bleeding.

On day two, the patient went into acute hypoxemic respiratory failure due to PE requiring intubation. She subsequently developed shock and was started on norepinephrine for pressure support. On day five, she had melena, the bloody gastric residue was found in the nasogastric tube and hemoglobin dropped from 12.2 g/dL to 7.7 g/dL. Heparin was stopped, two units of blood were transfused, and an inferior venae cava (IVC) filter was placed. Upper endoscopy revealed extensive, circumferential deep ulceration in midesophagus with wall irregularity, friable, sloughy mucosa, and blackish discoloration of mucosa likely esophageal necrosis (Figures 2 and 3). Distal esophagus, gastro-esophagus junction, stomach, and duodenum appeared normal. Biopsies were not taken to avoid the risk of esophageal perforation. At this time, upon review, her prior CT chest showed a large TAA with mural thrombus causing compression in the midesophagus correlating with the area where on endoscopy was found to have a black esophagus. After consulting with the vascular surgery team, it was deemed that the patient is unstable to be considered for aneurysm repair or thrombectomy.

The patient was managed supportively with bowel rest, proton pump inhibitor (pantoprazole) infusion, IV fluids, antibiotics, and norepinephrine. While her hemoglobin was being stabilized, on day 23, due to the severity of the necrosis, deterioration of the overall condition, and persistent sepsis, she went into pulseless electrical activity and died after unsuccessful resuscitation.

## 3. Discussion

AEN, also referred to as “black esophagus” or “necrotizing esophagitis,” was first described in 1990 by Goldenberg [8]. After that, several case reports have described this finding in critically ill patients with comorbidities. Even though distal esophageal necrosis is common due to its vasculature, midesophageal necrosis has also been reported usually due to compression by hematoma formed after TAA rupture or after its repair or aortic dissection.

Watanabe et al. [9] described a case of esophageal necrosis secondary to ruptured thoracic aortic aneurysm resulting in mediastinal hematoma causing compression of the esophagus. Several other case reports of esophageal necrosis have been described after thoracic endovascular aortic repair (TEVAR) due to mediastinal hematoma (Tobisch et al. [10], Koizumi et al. [11], Abou-Al-Shaar et al. [12], Seto et al. [13]). In contrast to these cases, in our case, there was no evidence of rupture of TAA or mediastinal hematoma. The possible explanation for esophageal necrosis in our case is due to direct extrinsic esophageal compression secondary to enlarged aortic aneurysm (Figure 1), which can obliterate local venous return and arterial blood supply leading to infarction [14]. It is unlikely that a single etiological factor is responsible for this condition. The accepted hypothesis is a two-hit hypothesis. In our case, a decreased blood supply to the midesophagus by extrinsic compression of TAA is further exacerbated by low flow vascular state, hypotension from PE.

Endoscopy is the gold standard for diagnosing AEN. It appears as a circumferential black discoloration with underlying friable hemorrhagic tissue and a sharp transition to normal-appearing mucosa at the GEJ [5]. Although proximal involvement has been described, AEN usually involves the distal third of the esophagus, as it is the least vascular area in the esophagus. However, the involvement of midesophagus without distal esophagus is rare, which occurred in our case as a result of compression of TAA in the midesophagus and also decreased blood supply from esophageal arterial branches of the thoracic aorta due to mural thrombus in the TAA.

Staging of AEN based on endoscopy findings is mentioned in Table 1.

Biopsy differentiates AEN from other conditions such as malignant melanoma, acanthosis nigricans, coal dust deposition, pseudomelanosis, and melanocytosis of the esophagus [15, 16]. Biopsy is usually recommended but not required for the diagnosis [8].

AEN generally carries a poor prognosis with a mortality rate of 32% as these patients have multiple comorbidities like in our case. Esophageal perforation occurs as a complication in <7% of patients with AEN and requires urgent surgical intervention [2]. Other complications include mediastinitis and abscess formation. Esophageal stricture is a long-term complication likely to result from protective scar formation which has been reported in 25–40% of patients, common in stage 2 and 3 of AEN, and requires endoscopic dilatation [2].

Treatment mainly includes supportive care to increase organ perfusion with IV hydration, optimization of acid suppression using intravenous proton pump inhibitors, and treatment of underlying illness. Oral intake should be avoided for at least 24 hours, after which sucralfate suspension can be considered for its cryoprotective effects and its ability to bind pepsin and stimulate mucus secretion [17]. Empirical antibiotics should be initiated in suspected cases of esophageal perforation, unexplained fevers, rapid clinical decompensation, and immunocompromised individuals as in cirrhosis, AIDS, transplant recipient, and dialysis patients [18]. Surgical intervention in patients with AEN is reserved for perforated esophagus with resultant mediastinitis and abscess formation [19].

## 4. Conclusion

In summary, we described a case of esophageal necrosis as a result of a low flow state from PE (initial insult) that was exacerbated by compression from unruptured TAA in a critically ill patient and presented as upper GI bleed. AEN should be considered as one of the differential diagnosis of upper gastrointestinal bleed, especially in elderly patients with multiple comorbidities and hypotension in critically ill patients. Early diagnosis and management of AEN are necessary to prevent complications and to improve patient's outcome.

## Figures and Tables

**Table 1:** Staging of AEN based on endoscopy findings.

Stage	Endoscopy findings

Stage 0	Prenecrotic viable esophagus
Stage 1	Diffuse, circumferential, black-appearing esophageal mucosa with occasional yellow exudates
Stage 2	Residual black areas in the esophagus, thick white exudates composed of necrotic debris covering friable pink mucosa
Stage 3	Normal endoscopic appearance, granulation tissue presents only microscopically

## Data Availability

No data were used to support this study.
